# Up-regulation of DNA2 results in cell proliferation and migration in endometriosis

**DOI:** 10.1007/s10735-021-09983-z

**Published:** 2021-05-28

**Authors:** Xinyan Wang, Wenjie Zeng, Sheng Xu, Jingya Nie, Lu Huang, Yucheng Lai, Yan Yu

**Affiliations:** 1 Department of Gynecology, Zhejiang Provincial People’s Hospital, People’s Hospital of Hangzhou Medical College, Hangzhou, China; 2 Department of Gynecology, Nanxun People’s Hospital, Huzhou, China

**Keywords:** Endometriosis, DNA replication ATP-dependent helicase/nuclease 2, DNA2, Endometrial mesenchymal stem cells, Checkpoint kinase 1, CHK1

## Abstract

Accumulating evidence has suggests that women with advanced endometriosis exhibit alterations in the expression of genes in the endometrium compared to healthy controls. Furthermore, replication stress is a characteristic feature of cancer cells, which results from sustained proliferative signaling induced by either the activation of oncogenes or the loss of tumor suppressors. In the present study, we propose that DNA replication ATP-dependent helicase/nuclease 2 (DNA2) might be upregulated in endometriosis. Immunohistochemical staining results confirmed the hypothesis that DNA2 is overexpressed in the eutopic/ectopic endometrium compared to that in a control endometrium from a healthy donor. Subsequently, ectopic endometrium-derived endometrial mesenchymal stem cells (EMSCs) showed the highest level of DNA2 and checkpoint kinase 1 (CHK1), as well as the strongest proliferation and migration capabilities, followed by eutopic endometrium-derived EMSCs, and then control EMSCs. To further analyze the function of DNA2, we knocked-down DNA2 expression in KLE cells. As expected, proliferation and migration declined when cells were transfected with DNA2 small interfering RNA. Taken together, our study demonstrated the overexpression of DNA2 in human endometriosis, which might be responsible for the upregulated cell proliferation and migration. This study provides insights into the mechanisms underlying human endometriosis.

**Supplementary Information:**

The online version contains supplementary material available at 10.1007/s10735-021-09983-z.

## Introduction

Endometriosis (EM) is an estrogen-dependent benign inflammatory response disease and refers to the appearance of endometrial interstitials and glands outside the uterine cavity (Burney and Giudice 2012; Giudice 2010). Common ectopic endometrial tissue invasion sites include the ovary, peritoneum, uterosacral ligament, and recto-uterine pouch (Keckstein and Wiesinger 2005; Kinkel et al. 1999; Ness 2003; Young et al. 2013). The incidence of this disease has shown an upward trend in recent years. Furthermore, 10–15% of women of childbearing age suffer from the disease, of which 30% present with infertility and 45% of cases had chronic pelvic pain (Kulkarni 2016; Nnoaham et al. 2011). Studies have also reported a high recurrence rate of 20–40% after treatment (Becker et al. 2017; Fedele et al. 2004; Tandoi et al. 2011). Therefore, there is a need to improve our understanding of the pathogenesis of endometriosis to develop more clinically effective therapies.

There are many theories for the pathogenesis of endometriosis (Koninckx et al. 2019; Sourial et al. 2014). However, Sampson's theory of retrograde menstruation/transplantation is the most popular and accepted mechanism (Signorile and Baldi 2010). Moreover, accumulating evidence suggests that endogenous or exogenous estrogen plays an important role in the pathogenesis of endometriosis (Burney and Giudice 2012; Han et al. 2015). Estrogen regulates various types of cells in the endometrium, including epithelial cells, stromal cells, immune cells, and vascular cells. Therefore, the anabolic or regulatory imbalance of estrogen can cause pathological changes in the endometrium (Gibson et al. 2015; Skarzynski et al. 2020; Xu et al. 2019; Yang et al. 2018). Enzymes related to estrogen anabolism have been observed in endometriosis tissues (Dassen et al. 2007; Lai et al. 2019; Tang et al. 2019).

Estrogen can stimulate cell proliferation and induce DNA damage (Roy et al. 2007; Santen et al. 2015). Many studies have reported that DNA replication helicase/nuclease 2 (DNA2) is overexpressed in tumors, such as breast and ovarian cancers (Strauss et al. 2014). DNA2 is a key enzyme, with both helicase and nuclease activity, and is involved in both DNA replication and DNA repair in the nucleus and mitochondrion (Pawłowska et al. 2017; Ronchi et al. 2019; Yu et al. 2018). In this study we sought to determine whether DNA2 is involved in endometriosis.

## Material and methods

### Ethics approval and human sample collection

This study was performed in accordance with the principles of the Declaration of Helsinki. Approval was granted by the Ethics Committee of Zhejiang Provincial People’s Hospital (November 9, 2019/No. 2019KY204). Human eutopic endometrial and ectopic endometriosis tissue samples were collected from patients with endometriosis, and endometrial samples were collected from control subjects undergoing laparoscopic surgery. Informed consent was obtained from all patients/subjects. All of the cases, including patients and fertile control subjects, were free from hormonal therapy for three months, and free from any other endocrine, metabolic, or immune diseases related to the female reproductive system before the collection of samples. There were no obvious abnormalities in the heart, lung, liver, kidney, or other functional examinations. All healthy control subjects were nulligravid.

### Isolation and culture of endometriosis cells

Primary endometrial cells were isolated from eutopic/ectopic endometrium and normal human endometrial tissues within 2 h of separation. Briefly, the fresh tissues were rinsed thrice with phosphate-buffered saline (PBS, BBI life Sciences) and divided into two parts. One part was preserved with tissue specimen fixative solution for immunohistochemistry (IHC) study, and the other was cut into 0.5–1.0 mm pieces with ophthalmic scissors in a cell culture dish. Then, three times the volume of pre-warmed 0.25% collagenase IV (Sigma) /trypsin–EDTA (GIBCO) mixed solution was added, and the mixture was placed in a 37 °C cell incubator. After 10 min, two volumes of culture medium (DMEM-F12 supplemented with 10% FBS; GIBCO) was added to stop the digestion. The cell suspension was filtered through a mesh and centrifuged at 700 rpm for 3 min. The pellets were resuspended in culture medium at a density of 1 × 10^5^ cells/mL and seeded in 10-cm culture dishes at 37 °C, 5% CO_2_, and passaged by 0.25% trypsin–EDTA digestion at a 1:3 ratio.

The phenotype of endometrial cells was examined at the third passage by flow cytometry (BD FACSVerse) with antibodies, including anti-CD14 (BD Biosciences), anti-CD34 (BD Biosciences), anti-CD73 (Biolegend), anti-CD105 (Biogems), anti-CD45 (BD Biosciences), and anti-CD90 (Biolegend) according to manufacturer’ instructions. The suitable isotype-matched antibody (BD Biosciences) was utilized as the negative control. The data were analyzed using BD FACSuite software.

### Histopathological examination of DNA2

Eutopic/ectopic endometrium and normal human endometrial tissues were fixed in paraphormaldeyde (Sangon Biotech) and embedded in paraffin. The 5 μm specimen sections were cut, mounted on glass and dried overnight at 37 °C. All sections were then deparaffinized in xylene, rehydrated through a graded alcohol series and washed in PBS. For IHC staining, the tissue sections were first incubated with sodium citrate buffer for antigen retrieval and incubated for 30 min in blocking solution followed by an overnight incubation with the primary antibody against DNA2 (Abcam). The tissue sections were then sequentially incubated with a biotinylated antibody and peroxidase-labeled streptavidin (Dako), resulting in a brown precipitate at the antigen site.

### Cell lines and reagents

The endometrial adenocarcinoma cell line KLE was obtained from the American Type Culture Collection, and maintained in MEM medium supplemented with 10% fetal bovine serum (FBS), 100 µg/mL streptomycin, 100 units/mL penicillin, and 2 mM glutamine (GIBCO). Cell cultures were maintained at 37 °C in an atmosphere containing 5% CO_2_ and 100% humidity.

β-Estradiol (E2), progesterone, tamoxifen, and monoclonal antibodies for ER-β (SAB4500814) and goat anti-rabbit secondary antibody (A4062) were purchased from Sigma (St. Louis, MO, USA). Antibodies against ER-α (MC-20), PR-A and PR-B (C-20) were purchased from Santa Cruz Biotechnology (Santa Cruz, CA, USA). Rabbit anti-β-actin antibody (TA306308) was purchased from Oncogene (Boston, MA, USA).

### Transfection of DNA2 small interfering (si) RNA

DNA2 siRNA and negative control (NC) were transfected into KLE cells or EMSCs isolated from ectopic endometrium using standard transfection reagent (Thermo Scientific) according to the manufacturer’s protocol, to observe the effect of DNA2 expression levels on the cell phenotype.

### RNA extraction, reverse transcription, and quantitative real-time polymerase chain reaction (qRT-PCR)

Total RNA was isolated using Trizol reagents (TAKARA) according to the manufacturer’s protocol. cDNA was synthesized from 1 µg RNA with random primers using the SuperScript kit (Invitrogen). RT-qPCR was performed in the ABI 7900 real-time RT-PCR system with reagents from the SYBR® Green Real-time PCR Master Mix (Takara) and the appropriate primers in a 20 μL reaction system (10 μL Master mixture, 1 μL cDNA, 1 μL forward primer and 1 μL reverse primer, and 7 μL ddH_2_O). The primers for each gene were as follows: DNA2, forward TTTTGTATTGTGGATGAAGCCTCT, reverse CATTCTGTACTGCACGGTTAACTG; CHK1, forward GCTGATTGATATTGTGAGCAGCC, reverse TTCATCCTTTCCCCAAAGTTTTG; and GAPDH, forward GGCACAGTCAAGGCTGAGAATG, reverse ATGGTGGTGAAGACGCCAGTA. Real-time PCR was performed under the following conditions: initial denaturation at 95 °C for 10 min, followed by 40 cycles of denaturation at 95 °C for 30 s, annealing at 60 °C for 40 s, and extension at 72 °C for 30 s. All experiments were performed at least three times and the mean values were used.

### Western blot analysis

Western blot analysis was performed as previously described. In brief, cell extracts (20 µg) were separated on 10% SDS PAGE (BBI life Sciences) and subsequently transferred onto nitrocellulose membranes (Millipore). The membranes were blocked for 1 h in PBS containing 0.1% Tween-20 and 10% nonfat dried milk. The specific antibodies against ER-α, ER-β, PR-A, PR-B, or β-actin were applied according to the manufacturer’s recommendations. Primary antibody binding was performed overnight at 4 °C with constant rotation. The blots were then incubated with appropriate secondary antibodies (at room temperature for 1 h at 1:3000 dilution) and developed with an enhanced chemiluminescence kit (Beyotime).

### Proliferation assay

Cell proliferation was determined using the Cell Counting Kit-8 (CCK-8) (Beyotime) according to the manufacturer’s protocol. Cells were seeded in 96-well plates at a confluence of 2000 cells per well. The proportion of living cells was measured at indicated time points by absorbance at 460 nm using a microplate reader according to the manufacturer’s instructions.

### Migration assay

Transwell inserts (Millipore) were used for the analysis. 5 × 10^4^ cells were seeded onto the upper chamber, and 800 μL medium with 10% FBS was added to the lower chamber. After incubation for 24 h, the cells adhering to the upper surface of the membrane were removed with a cotton swab. Migration cells, which adhered to the lower surface, were fixed with 4% paraformaldehyde and stained with 0.1% crystal violet (Beyotime). Data were obtained from three independent experiments.

### Cell cycle analysis

To determine the cellular proliferation rate, a propidium iodide (PI)-based cell cycle detection Kit (Beyotime) was used. Cells were plated at 1 000/cm^2^, and detached with 0.25% trypsin after 48 h. Following collection, the cells were washed twice with cold PBS and fixed with 70% ice-cold ethanol at 4 °C overnight. The cells were then collected via centrifugation, washed twice with PBS, and stained with propidium iodide at room temperature for 15 min in the dark. Cell cycle assays were performed using a flow cytometer (BD Biosciences). Each experiment was performed in triplicate.

### Statistical analysis

Data are expressed as the mean ± standard deviation (SD). Comparisons between groups were analyzed using the Student's *t* test or ANOVA. *P* values < 0.05 were considered statistically significant.

## Results

### Human eutopic/ectopic endometrium display accumulation of DNA2

**Fig. 1:**
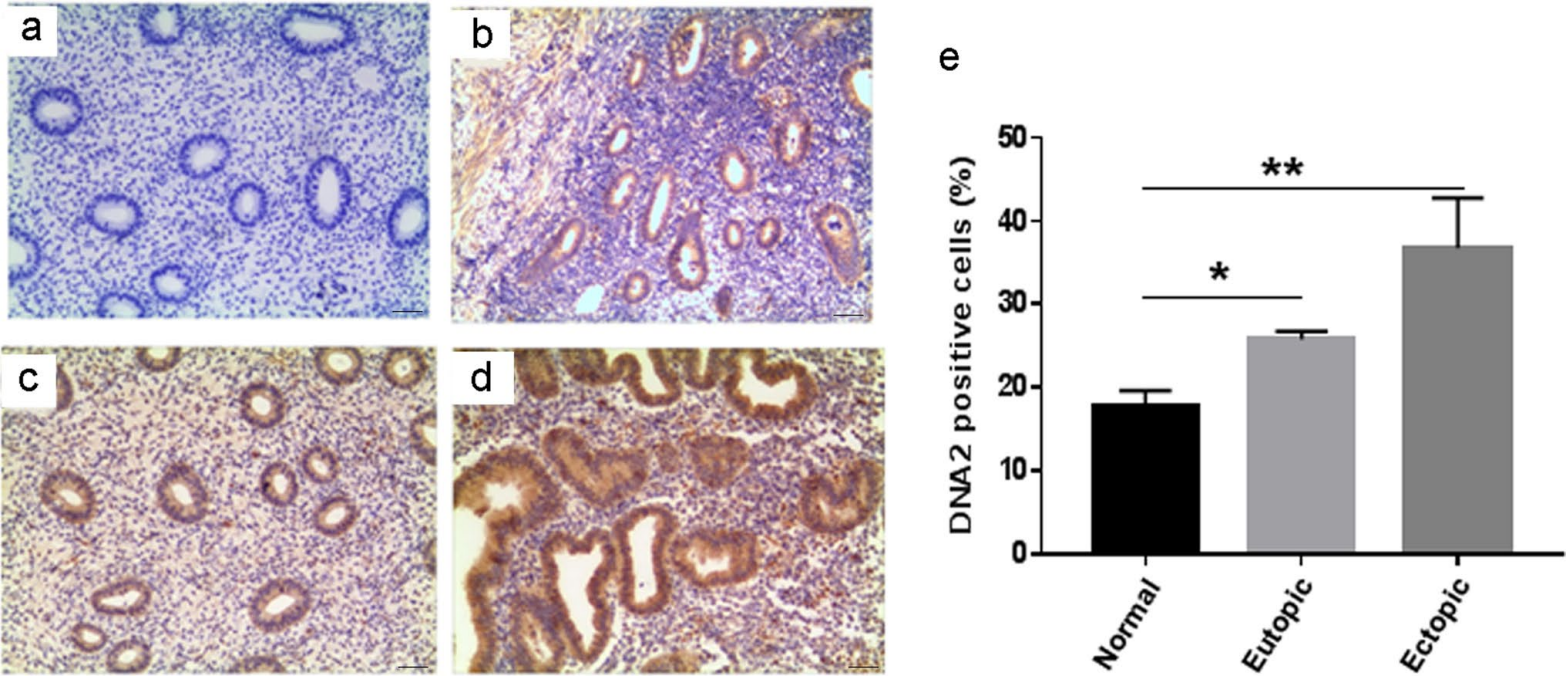
Eutopic/ectopic endometrium are enriched in DNA2. Staining distribution of DNA2 in representative tissue sections of negative control (**a**), normal endometrial tissues (**b**), eutopic endometrium tissue (**c**), and ectopic endometrium tissue (**d**). Images are representative of n = 10. The brown precipitate indicates the antigen site. **e** DNA2 positive cells were analyzed using InForm Version 1.0 software (INFORM). Five images per slide were quantified. (*P < 0.05, **P < 0.01). Scale bars = 50 μm. *DNA2* DNA replication ATP-dependent helicase/nuclease 2

**Fig. 2:**
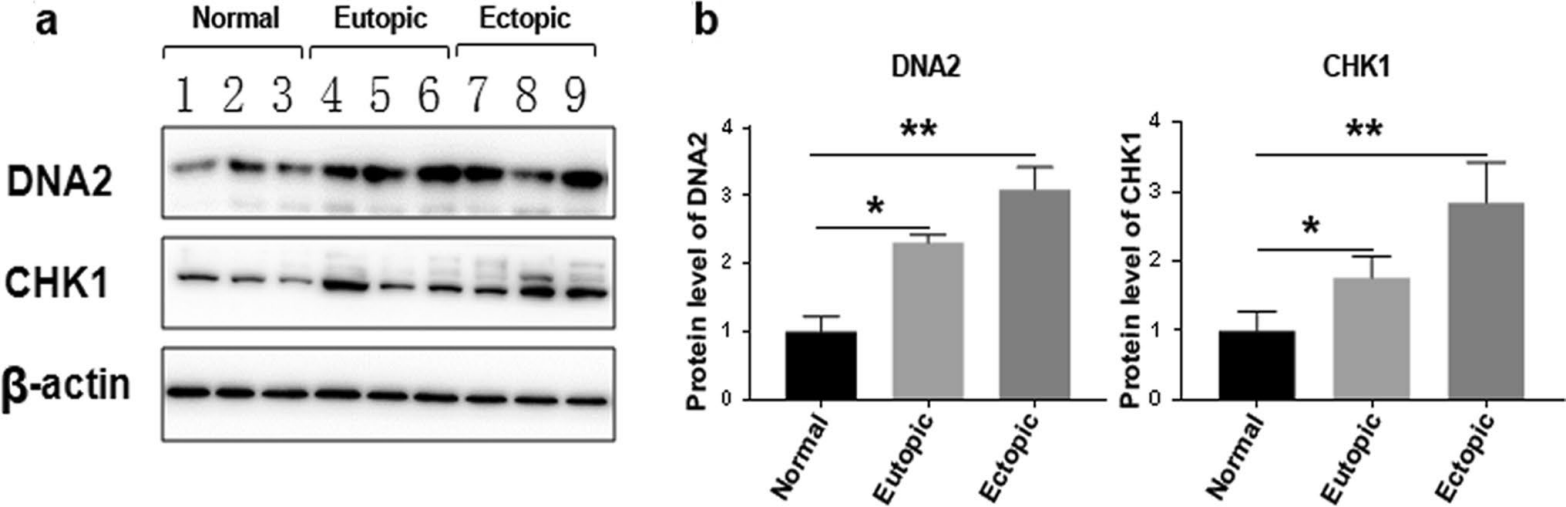
DNA2 and CHK1 expressions in eutopic/ectopic endometrium and normal human endometrial tissues. **a** Protein expression of DNA2 and CHK1 in endometrial tissues were determined by Western blot analysis. **b** Quantification of DNA2 and CHK1 levels were determined using image J software. (*P < 0.05, **P < 0.01). *DNA2* DNA replication ATP-dependent helicase/nuclease 2; *CHK1* checkpoint kinase 1

Disruption of DNA2 has been associated with many types of human diseases (Zheng et al. 2020). To clarify the role of DNA2 in endometriosis, we first assessed the levels of DNA2 in tissue samples obtained from patients with endometriosis. Of the 10 patients, 5 had an ectopic endometrium and the others had eutopic endometrium. The mean age and body mass index (BMI) of the patient group were 31.1 ± 3.6 years and 23.8 ± 2.2 kg/m^2^, respectively. Matched human endometria (n = 5) were collected from the control women. The mean age and BMI of the controls were 31.8 ± 1.8 years and 24.1 ± 2.6 kg/m^2^, respectively. As shown in Fig. 1, we subsequently investigated DNA2 expression using IHC. For all cases of control endometrium, IHC of tissue sections revealed that DNA2 staining ranged in intensity from mild to moderate (≈ 18% cells stained positive). Among the endometriosis samples tested, DNA2 staining varied from mild to strong and was more evenly distributed (> 30% cells stained positive). Having confirmed that DNA2-positive cell ratios were increased in endometriosis tissues, we investigated the mRNA and protein levels in these tissues. Upregulated levels of DNA2 were observed by Western blotting (Fig. 2). Moreover, we evaluated CHK1, which is also an essential kinase required to preserve genome stability; similar results were obtained in the present study. Together, these data suggest that both CHK1 and DNA2, two key players in DNA damage repair, were upregulated in endometriosis samples.

### Endometrial cells isolated from eutopic/ectopic endometrium tissues showed enhanced proliferation and migration capability

**Fig. 3:**
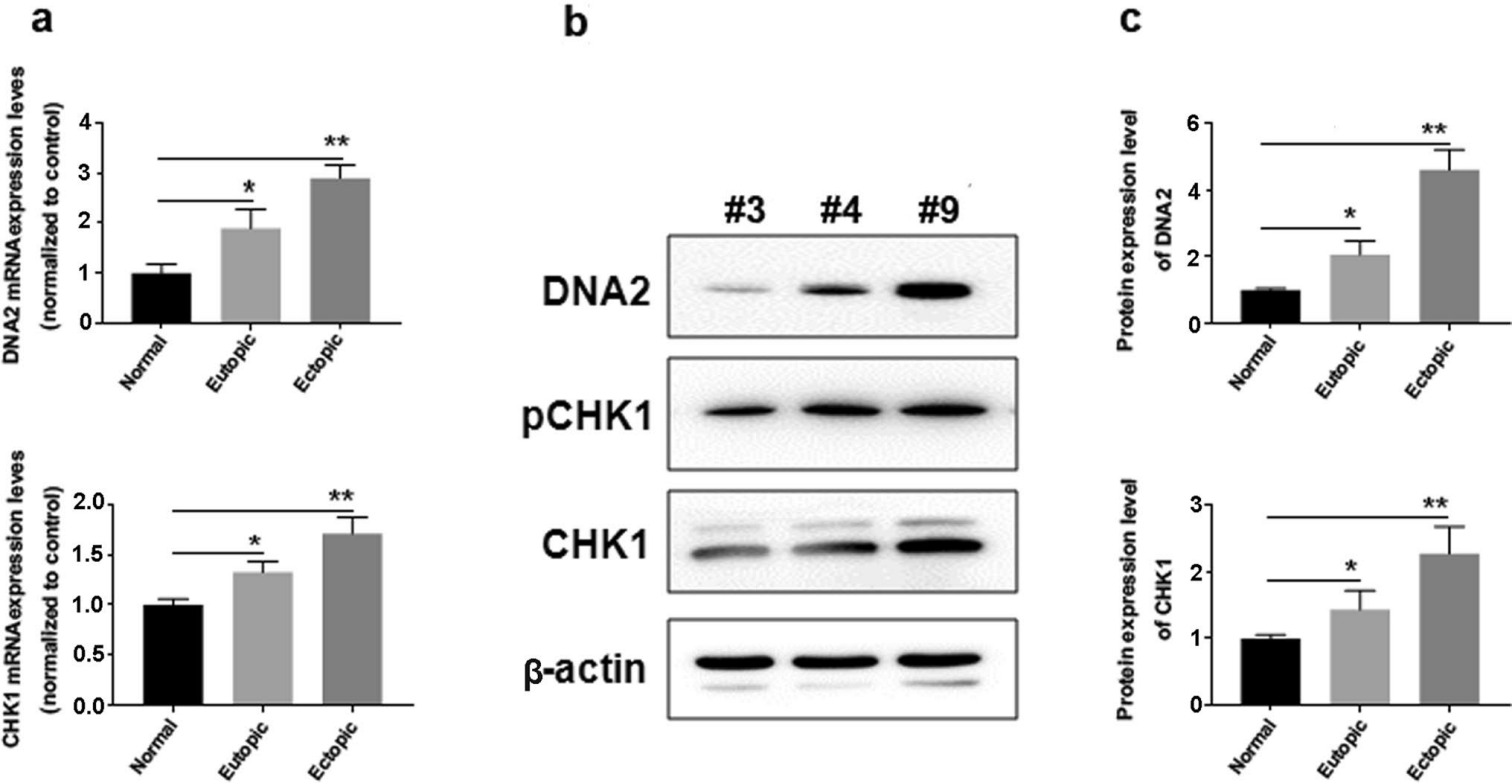
DNA2 and CHK1 expressions in three types of primary endometrial cells. Primary endometrial cells were isolated from eutopic/ectopic endometrium and normal human endometrial tissues, respectively. **a** qPCR revealed that DNA2 mRNA expression was upregulated in eutopic/ectopic endometrium-derived endometrial cells compared with those from normal endometrial tissues. A similar expression trend of CHK1 was also observed. **b**, **c** Western blot analysis results showed 1 representative cell line per group, which confirmed the enhanced expression of DNA2 and CHK1. Correspondingly, pCHK1 level was significantly higher in eutopic/ectopic endometrium-derived endometrial cells. (*P < 0.05, **P < 0.01). *DNA2* DNA replication ATP-dependent helicase/nuclease 2; *CHK1* checkpoint kinase 1

Typical EMSCs were isolated from all three groups using a collagenase IV/trypsin–EDTA-based protocol. Primary EMSCs presented a fusiform morphology, which gradually changed into a fibroblast-like spindle shape with increasing passages, similar to the features of bone marrow MSCs. All EMSCs positively expressed CD73, CD90, and CD105 but negatively expressed CD14, CD34, and CD45 (Supplementary Fig. 1). Subsequently, EMSCs at passage 3 of each group were harvested and tested for CHK1 and DNA2 expression levels. At the mRNA levels, eutopic endometrium-derived EMSCs showed an ≈ 1.2-fold increase in CHK1 and ≈ 1.9-fold increase in DNA2 levels compared with control EMSCs (Fig. 3a), which was further improved in the ectopic endometrium-derived EMSCs (≈ 1.5-fold increase in CHK1 and ≈ 2.7-fold increase in DNA2 levels). Representative EMSCs of each group (controls: #3; eutopic endometrium group: #4; ectopic endometrium: #9) were analyzed by Western blotting (Fig. 3b, c). In addition, enhanced phosphorylation of CHK1 was also observed in EMSCs from patients with endometriosis.

**Fig. 4:**
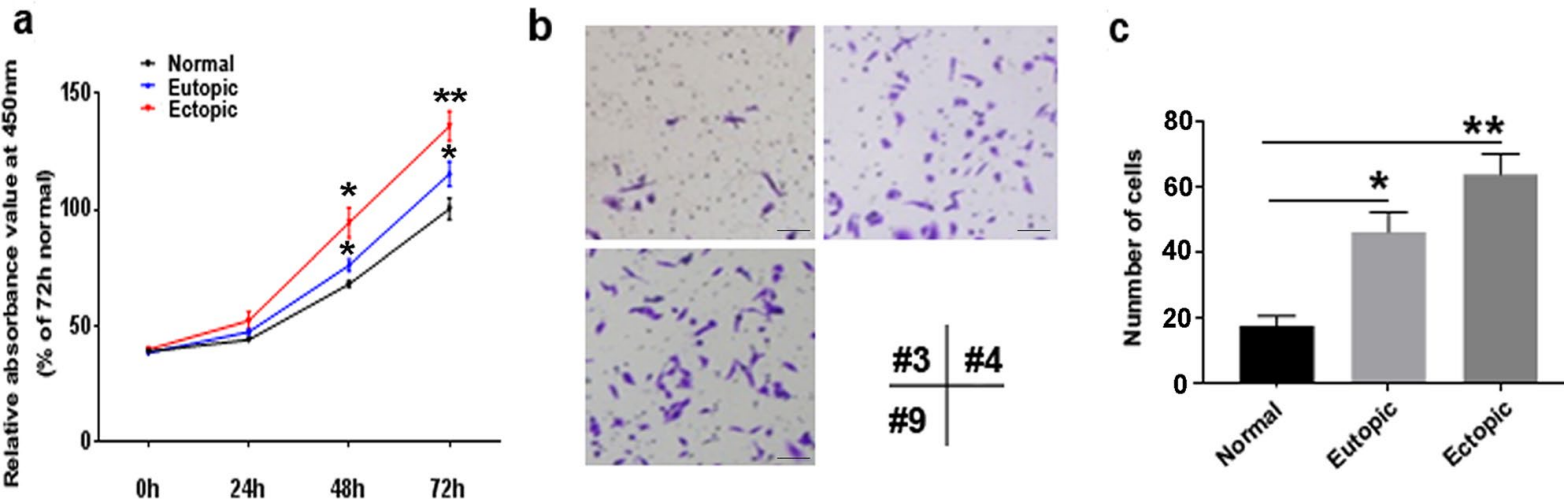
Endometrium-derived endometrial cells show enhanced proliferation and migration capability. **a** Cell Counting Kit-8 assay revealed that eutopic/ectopic endometrium-derived endometrial cells grow faster than those from normal endometrial tissues. **b** Transwell migration assay. Representative microscopic images of cells that migrated through the Transwell in the migration assay (crystal violet staining). **c** The quantitation of cells that migrated through the Transwell in the migration assay. The data are presented as the mean number of migrated cells per visual field. (*P < 0.05, **P < 0.01). Scale bars = 50 μm

The CCK-8 assay results indicated EMSCs from patients with endometriosis exhibited significantly increased proliferation compared with the controls (Fig. 4a). Furthermore, the Transwell assays revealed the number of migrated cells was significantly increased in both the eutopic endometrium-derived EMSCs (≈ 2.2-fold higher) and ectopic endometrium-derived EMSCs (≈ 3.2-fold higher) compared to controls. Moreover, migration of EMSCs isolated from ectopic endometrium was significantly reduced by DNA2 knockdown using siRNA (Supplementary Fig. 2). These results suggest a potential function of DNA2 in EMSC migration and endometriosis.

### E2 treatment upregulates DNA2 and CHK1 expressions in endometrial cells

**Fig. 5:**
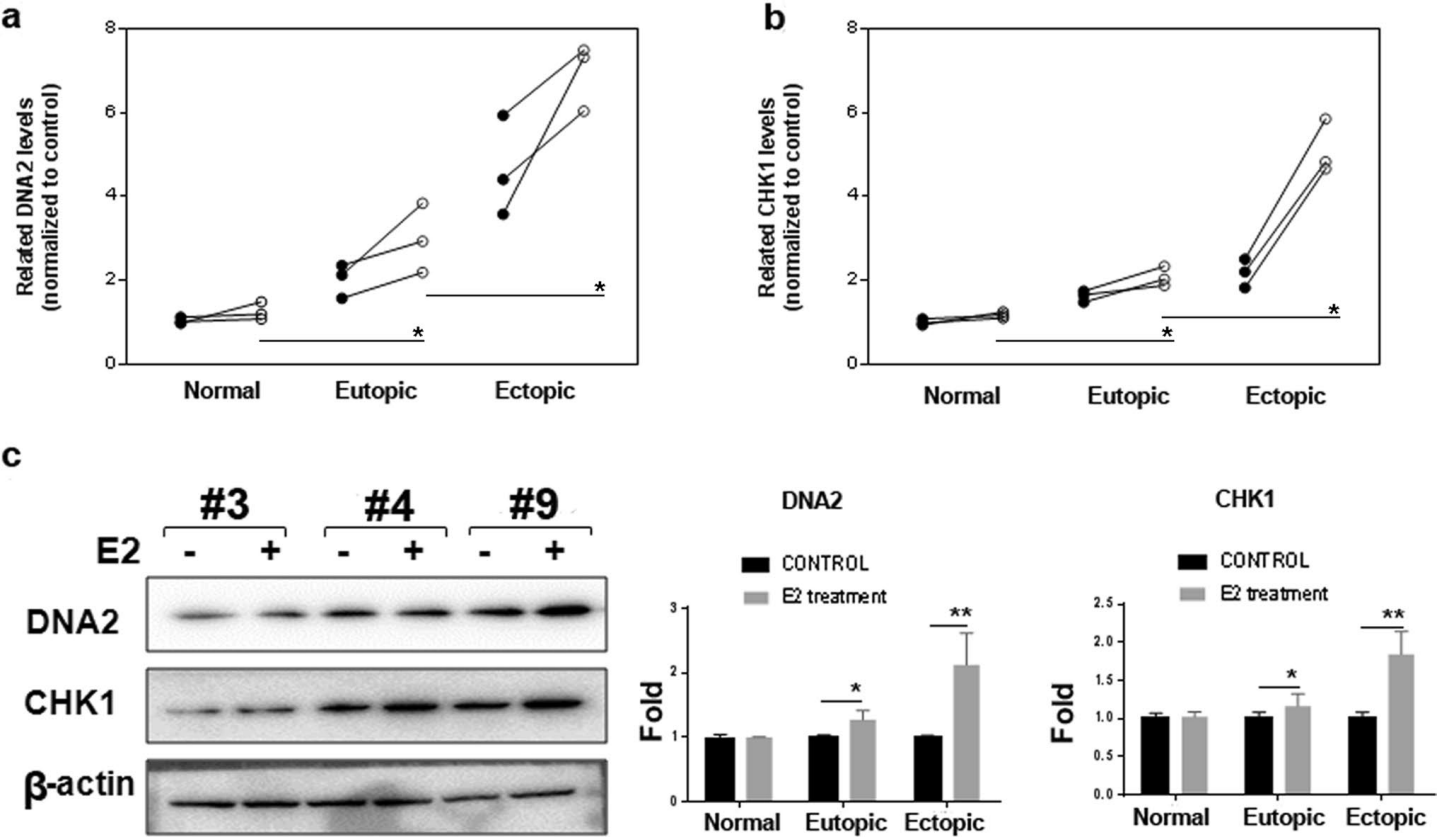
Effect of E2 treatment on DNA2 and CHK1 expressions in three types of primary endometrial cells. In vitro cultured endometrial cells were treated with/without E2, and harvested after 24 h for the mRNA and Western blot analysis. **a**, **b** DNA2 and CHK1 mRNA levels were significantly increased in eutopic/ectopic endometrium-derived endometrial cells, which was more obvious in the ectopic endometrium-derived group. **c** Western blot analysis results were consistent with the qPCR data. (*P < 0.05, **P < 0.01). *DNA2* DNA replication ATP-dependent helicase/nuclease 2; *CHK1* checkpoint kinase 1; *E2* estradiol

Subsequently, we proposed that endometrial cells from patients with endometriosis might be more sensitive to E2 treatment. To verify this hypothesis, EMSCs at passage 4 in all three groups were administered E2. As shown in Fig. 5, there were no significant changes in the DNA2 and CHK1 levels in the controls. Interestingly, EMSCs from patients with endometriosis were sensitive to E2 treatment, resulting in a 1.5–2-fold increase in DNA2 mRNA expression and 1.2–2.8-folds increase in CHK1 mRNA levels (Fig. 5a, b). This was also confirmed by Western blot analysis (Fig. 5c).

### Knock down of DNA2 inhibited cell cycle, proliferation and migration in endometrial cell line

**Fig. 6:**
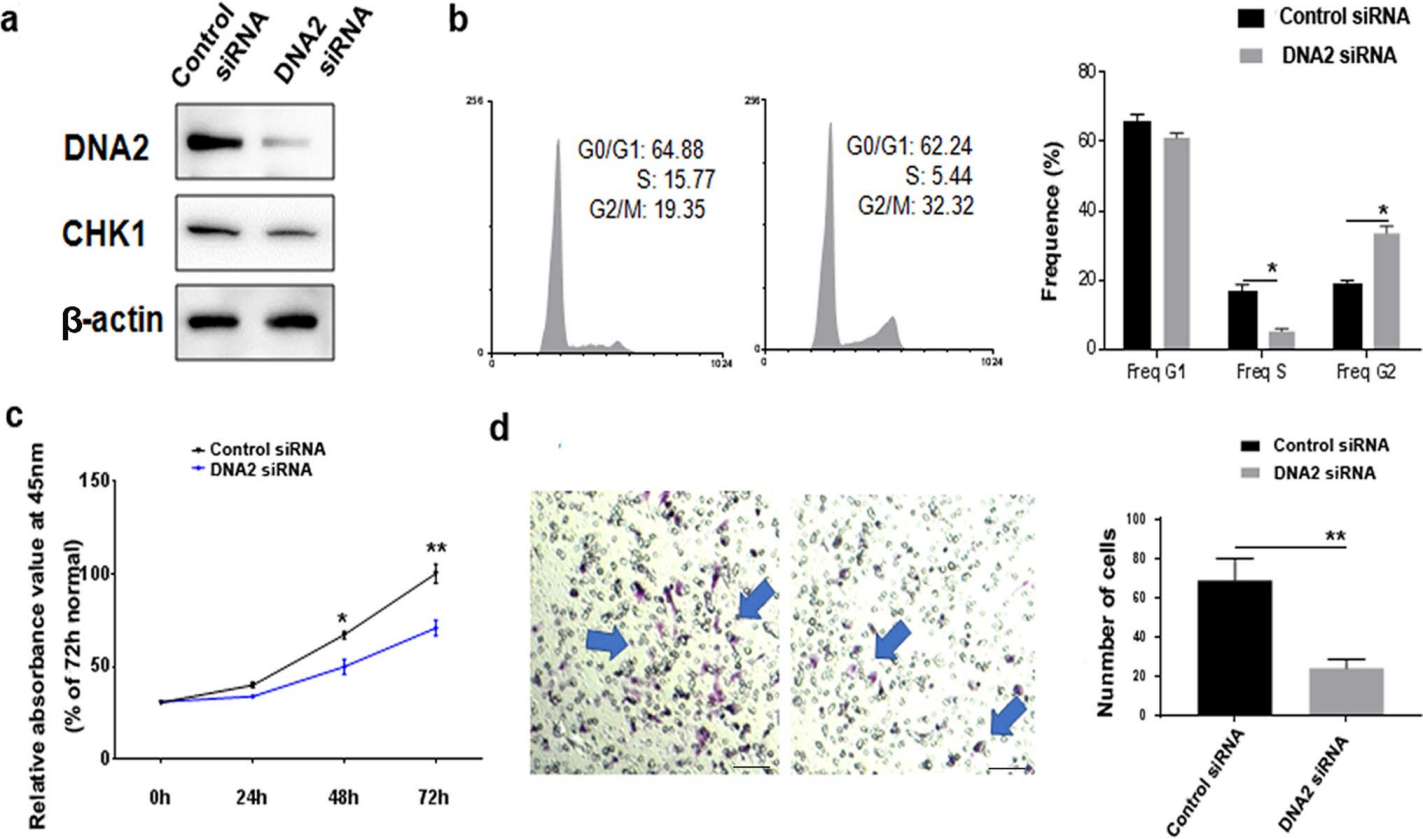
DNA2 knockdown inhibits the growth and migration of endometrial adenocarcinoma cell line in vitro. **a** DNA2 expression was significantly downregulated in KLE cells following transfection with the DNA2 siRNA compared with the control siRNA. The expression of DNA2 and CHK1 was normalized to beta-ACTIN. **b** DNA2 knockdown inhibits the cell cycle of KLE cells. FCM was used to detect the cell cycle. Cells transfected with DNA2 siRNA accumulated in the G2/M phase. **c** Proliferation of KLE cells was inhibited after DNA2 siRNA administration. **d** KLE cells transfected with the DNA2 siRNA decreased cell migration compared with siRNA control. (*P < 0.05, **P < 0.01). Scale bars = 50 μm. *DNA2* DNA replication ATP-dependent helicase/nuclease 2; *CHK1* checkpoint kinase 1

To further analyze the role of DNA2 in proliferation and migration in endometrial cells, we treated the KLE endometrial cells with DNA2 siRNA. As shown in Fig. 6a, DNA2 levels were significantly reduced 36 h after transfection with specific DNA2 siRNA; the CHK1 protein levels were not significantly changed. Cell cycle assays showed that KLE cells treated with DNA2 siRNA had a significantly reduced cell population in the S phase and increased cell populations in the G2 phase compared with the siRNA controls (Fig. 6b). Subsequently, cell growth was measured using the CCK8 assay, and migration was determined by Transwell assay. As expected, proliferation and migration declined when cells were transfected with DNA2 siRNA (Fig. 6c, d).

## Discussion

Sampson's theory of retrograde menstruation/transplantation remains the most popular and accepted pathogenetic mechanism of endometriosis (Signorile and Baldi 2010); however, several lines of clinical and experimental evidence seem to contradict this hypothesis (Redwine 2002). Over the last decade, many studies have suggested that the environmental disruption of hormones, such as estrogen, resulted in endometriosis (Marquardt et al. 2019; Wagner and Lehmann 2006). Estrogen is an important mediator of endometrial homeostasis, and its anabolic or regulatory imbalances can cause pathological changes in the endometrium. High levels of estrogen have been shown to induce the generation of prostaglandins, which in turn stimulate cyclooxygenase 2, promote the expression of aromatase, and establishe a positive feedback to further enhance the production of estrogen (Bulun et al. 2002; Lai et al. 2019; Tamura et al. 2004). The endometrial glands contain 17β-Hydroxysteroid dehydrogenase type 2 (17HSD2), which causes the inactivation of E2 (Aka et al. 2010; Husen et al. 2001; Zeitoun et al. 1998). However, ectopic endometrial glands do not have 17HSD2, leading to a defect in the inactivation of E2. In contrast, they contain 17βHSD type1, which can promote the conversion of estrone to more effective E2 (Hudelist et al. 2007; Zeitoun et al. 1998). Thus, abnormally expressed enzymes in ectopic endometrial glands result in estrogen aggregation and ectopic endometrial proliferation.

In the present study, we found that human eutopic/ectopic endometrium displayed an accumulation of DNA2. These data were consistent with the findings of multiple tumor studies that showed that DNA2 is overexpressed, which could be the result of increased tolerance to replication stress by the activation of an oncogenic factor. Subsequently, we isolated EMSCs from both the control and eutopic/ectopic endometrial tissues. Multiple functional analysis data suggested that EMSCs derived from ectopic endometrium bear the highest levels of DNA2 and CHK1 as well as the strongest proliferation and migration capabilities. Moreover, eutopic/ectopic endometrium-derived EMSCs were highly sensitive to the E2 treatment, while the control EMSCs were not sensitive to the same dose of E2 administration. Finally, we used specific siRNA to knockdown the DNA2 expression in KLE cells and EMSCs from the ectopic endometrium. As expected, the proliferation and migration were declined when cells were transfected with DNA2 siRNA.

The findings of this study suggest that the temporary inhibition of DNA2 nuclease represents a promising strategy for the treatment of endometriosis. The next step will be to discover and test DNA2 inhibitors both in vitro and in vivo, which could be invaluable for endometriosis therapy either alone or in combination with other established strategies.

## Supplementary Information

Below is the link to the electronic supplementary material.

Supplementary file1 (TIF 1378 kb)
Supplementary Fig. 1 The phenotype of endometrial cells was examined at the third passage by flow cytometry.

Supplementary file2 (TIF 513 kb)
Supplementary Fig. 2 EMSCs isolated from ectopic endometrium were treated with DNA2 siRNA. a) At 36 h after DNA2 siRNA transfection, DNA2 protein levels were reduced by 70%, as revealed by Western blotting. b) DNA2 siRNA administration inhibited cell proliferation. c) Trans-well assay results suggested significantly reduced migration of DNA2-knock-down EMSCs compared to that of the controls. EMSCs, endometrial mesenchymal stem cells; DNA2, DNA replication ATP-dependent helicase/nuclease 2.

## Data Availability

The article contains all the data in the study.
