# Supplementary figures and images for: Up-regulation of DNA2 results in cell proliferation and migration in endometriosis
Source: J Mol Histol. 2021 May 28;52(4):741–9. doi: 10.1007/s10735-021-09983-z (PMC8324585; doi:10.1007/s10735-021-09983-z)

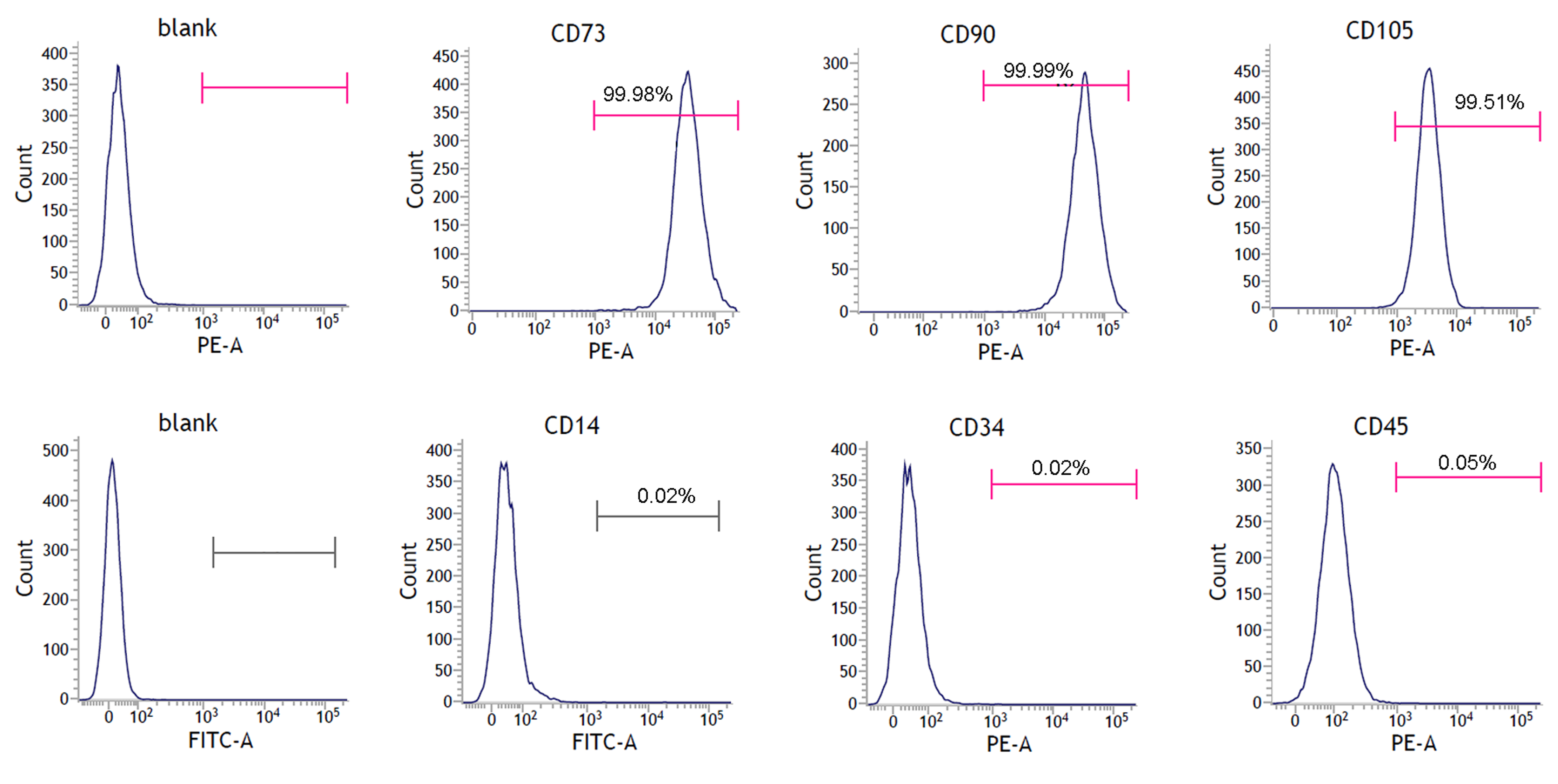

Supplement: Supplementary file 1 — Supplementary file1 (TIF 1378 kb) Supplementary Fig. 1 The phenotype of endometrial cells was examined at the third passage by flow cytometry. [file 10735_2021_9983_MOESM1_ESM.tif]

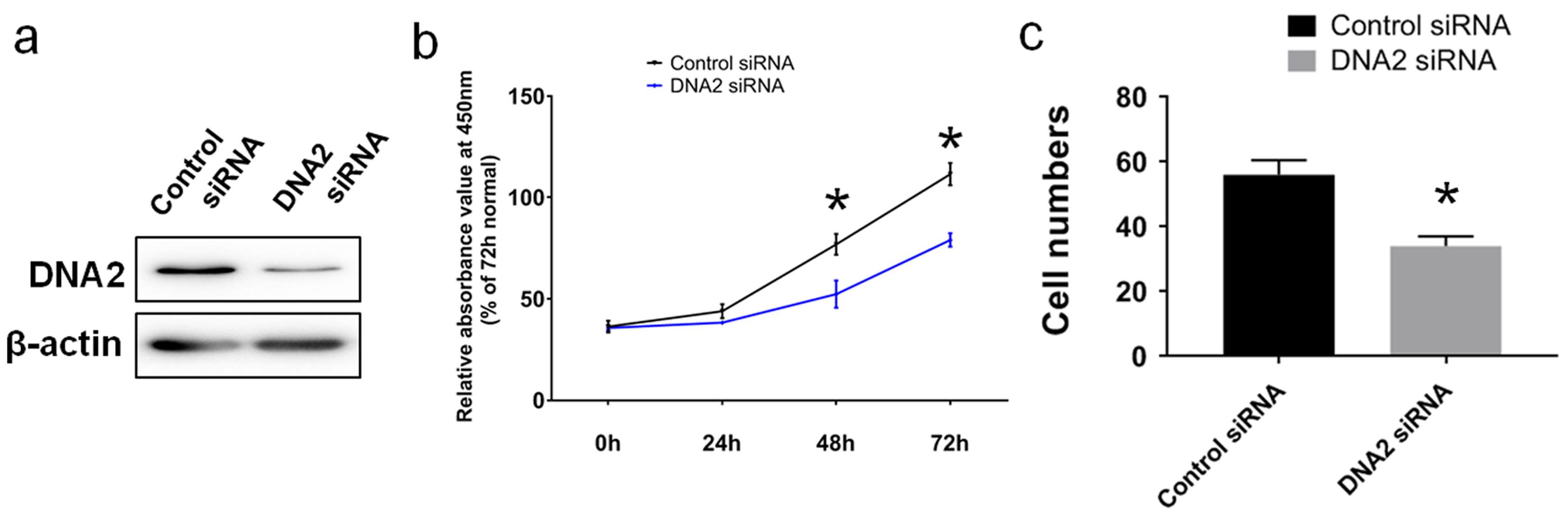

Supplement: Supplementary file 2 — Supplementary file2 (TIF 513 kb) Supplementary Fig. 2 EMSCs isolated from ectopic endometrium were treated with DNA2 siRNA. a) At 36 h after DNA2 siRNA transfection, DNA2 protein levels were reduced by 70%, as revealed by Western blotting. b) DNA2 siRNA administration inhibited cell proliferation. c) Trans-well assay results suggested significantly reduced migration of DNA2-knock-down EMSCs compared to that of the controls. EMSCs, endometrial mesenchymal stem cells; DNA2, DNA replication ATP-dependent helicase/nuclease 2. [file 10735_2021_9983_MOESM2_ESM.tif]
